# Supplementary material for: Effects of prenatal lead, mercury, cadmium, and arsenic exposure on children’s neurodevelopment in an artisanal small-scale gold mining area in Northwestern Tanzania using a multi-chemical exposure model
Source: PLOS Glob Public Health. 2025 Apr 30;5(4):e0004577. doi: 10.1371/journal.pgph.0004577 (PMC12043129; doi:10.1371/journal.pgph.0004577)
Supplement: S1 Table — Forward-backward stepwise regression performance metrics and variables maintained. The blue-colored variables are finally selected according to AIC and Deviance. (DOCX) [file pgph.0004577.s001.docx]

S1_Table: Forward-Backward Stepwise Regression performance metrics and variables maintained. The blue-colored variables are finally selected based on AIC and Deviance.

| Start: AIC = 434.22 |  | | |
| --- | --- | --- | --- |
| General ~ 1 |  |  |  |
|  | Df | Deviance | AIC |
| MUAC | 1 | 425.65 | 429.65 |
| Child.Age | 1 | 427.9 | 431.9 |
| Sex | 1 | 428.06 | 432.06 |
| geophagy | 1 | 429.06 | 433.06 |
| Mom.Education | 3 | 425.14 | 433.14 |
| <none> |  | 432.22 | 434.22 |
| Birth.Weight | 1 | 431.41 | 435.41 |
| Herbal | 1 | 431.53 | 435.53 |
| breastfeed_Birth | 1 | 432.21 | 436.21 |
| SEWQ.Status | 2 | 431.42 | 437.42 |
| MomOccupation | 2 | 431.54 | 437.54 |
|  | | | |
| Step: AIC = 429.65 |  |  |  |
|  | Df | Deviance | AIC |
| Mom.Education | 3 | 416.79 | 426.79 |
| Sex | 1 | 421.98 | 427.98 |
| geophagy | 1 | 422.41 | 428.41 |
| Child.Age | 1 | 422.91 | 428.91 |
| <none> |  | 425.65 | 429.65 |
| Birth.Weight | 1 | 424.05 | 430.05 |
| Herbal | 1 | 424.86 | 430.86 |
| breastfeed_Birth | 1 | 425.65 | 431.65 |
| MomOccupation | 2 | 425.46 | 433.46 |
| **SEWQ.Status** | **1** | **424.91** | **432.91** |
|  | | | |
| Step: AIC = 426.79 |  |  |  |
|  | Df | Deviance | AIC |
| Child.Age | 1 | 413.55 | 425.55 |
| Sex | 1 | 413.93 | 425.93 |
| <none> |  | 416.79 | 426.79 |
| geophagy | 1 | 414.88 | 426.88 |
| Birth.Weight | 1 | 415.56 | 427.56 |
| Herbal | 1 | 415.64 | 427.64 |
| MomOccupation | 2 | 414.42 | 428.42 |
| breastfeed_Birth | 1 | 416.75 | 428.75 |
| **Mom.Education** | **3** | **425.65** | **429.65** |
| - **SEWQ.Status** | 1 | 425.14 | 433.14 |
|  | | | |
| Step: AIC = 425.55 |  |  |  |
|  | Df | Deviance | AIC |
| Sex | 1 | 410.71 | 424.71 |
| <none> |  | 413.55 | 425.55 |
| Birth.Weight | 1 | 412.13 | 426.13 |
| geophagy | 1 | 412.15 | 426.15 |
| Herbal | 1 | 412.34 | 426.34 |
| **- Child.Age** | **1** | **416.79** | **426.79** |
| breastfeed_Birth | 1 | 413.46 | 427.46 |
| MomOccupation | 2 | 411.62 | 427.62 |
| **- Mom.Education** | **3** | **422.91** | **428.91** |
| - **SEWQ.Status** | 1 | 419.93 | 429.93 |
|  | | | |
| Step: AIC=424.71 |  |  |  |
|  | Df | Deviance | AIC |
| Birth.Weight | 1 | 408.57 | 424.57 |
| <none> |  | 410.71 | 424.71 |
| geophagy | 1 | 409.32 | 425.32 |
| Herbal | 1 | 409.54 | 425.54 |
| **- Sex** | **1** | **413.55** | **425.55** |
| **- Child.Age** | **1** | **413.93** | **425.93** |
| breastfeed_Birth | 1 | 410.63 | 426.63 |
| MomOccupation | 2 | 409.08 | 427.08 |
| **- Mom.Education** | **3** | **419.21** | **427.21** |
| -**SEWQ.Status** | 1 | 416.64 | 428.64 |
|  | | | |
| Step: AIC = 424.57 |  |  |  |
|  | Df | Deviance | AIC |
| <none> |  | 408.57 | 424.57 |
| **Birth.Weight** | **1** | **410.71** | **424.71** |
| Geophagy | 1 | 407.35 | 425.35 |
| Herbal | 1 | 407.58 | 425.58 |
| **Child.Age** | **1** | **412.05** | **426.05** |
| **- Sex** | **1** | **412.13** | **426.13** |
| breastfeed_Birth | 1 | 408.45 | 426.45 |
| **- Mom.Education** | **3** | **416.48** | **426.48** |
| MomOccupation | 2 | 407.18 | 427.18 |
| **- SEWQ.Status** | **1** | **415.35** | **429.35** |
