# Supplementary material for: Effects of prenatal lead, mercury, cadmium, and arsenic exposure on children’s neurodevelopment in an artisanal small-scale gold mining area in Northwestern Tanzania using a multi-chemical exposure model
Source: PLOS Glob Public Health. 2025 Apr 30;5(4):e0004577. doi: 10.1371/journal.pgph.0004577 (PMC12043129; doi:10.1371/journal.pgph.0004577)
Supplement: S2 Table — (DOCX) [file pgph.0004577.s002.docx]

S2_Table: Median Heavy metal levels by mother’s occupation

| **Mom Occupation** | **Cadmium** | **Lead** | **Mercury** | **Arsenic** |
| --- | --- | --- | --- | --- |
| Farming | 0.2 | 23.93 | 1.2 | 9.4 |
| Mining | 0.19 | 26.62 | 1.38 | 5.5 |
| Public Servant | 0.2 | 23.36 | 1.4 | 9.4 |
| chi-squared | 0.320 | 0.753 | 2.729 | 5.80 |
| *p*-value | 0.852 | 0.686 | 0.255 | 0.06 |
