# Supplementary material for: Effects of prenatal lead, mercury, cadmium, and arsenic exposure on children’s neurodevelopment in an artisanal small-scale gold mining area in Northwestern Tanzania using a multi-chemical exposure model
Source: PLOS Glob Public Health. 2025 Apr 30;5(4):e0004577. doi: 10.1371/journal.pgph.0004577 (PMC12043129; doi:10.1371/journal.pgph.0004577)
Supplement: Attachment — Submitted filename: PGPH-D-24-02727_reviewer.pdf [file pgph.0004577.s007.pdf]

PLOS Global Public Health

Effects of Prenatal Lead, Mercury, Cadmium, and Arsenic Exposure on Children's Neurodevelopment in an Artisanal Small-scale Gold Mining Area in Northwestern Tanzania Using a Multi-chemical Exposure Model

--Manuscript Draft--

**Manuscript Number:**	PGPH-D-24-02727
**Article Type:**	Research Article
**Full Title:**	Effects of Prenatal Lead, Mercury, Cadmium, and Arsenic Exposure on Children's Neurodevelopment in an Artisanal Small-scale Gold Mining Area in Northwestern Tanzania Using a Multi-chemical Exposure Model
**Short Title:**	Prenatal Exposure and Neurodevelopment in Mining Areas: A Multi-chemical Exposure Model
**Corresponding Author:**	Elias C. Nyanza, PhD<br>Catholic University of Health and Allied Sciences Bugando: Catholic University of Health and Allied Sciences<br>Mwanza, TANZANIA TANZANIA, UNITED REPUBLIC OF
**Order of Authors:**	Elias C. Nyanza
	Raphael J. Mhana
	Moses Asori
	Deborah S.K. Thomas
	Agapiti P. Kisoka
**Keywords:**	Neurodevelopmental; prenatal exposure; chemical mixture; gold mining; children
**Abstract:**	An estimated 200 million children under five fail to reach their cognitive development potential in Africa. Growing evidence suggests reduced neurodevelopment for children from environmental exposures, yet research on this topic in Sub-Saharan Africa remains limited. This study examined the effects of multi-chemical prenatal exposure to heavy metals on developmental milestones for children aged 3-4 in artisanal and small gold mining areas in northwestern Tanzania. This longitudinal follow-up study of children whose mothers were enrolled in the Tanzania Mining and Health Cohort in Geita District in 2015 was assessed at 3-4 years of age for the current study between June 2019 – June 2020. Developmental outcomes (cognitive, social, motor, and language skills) were assessed using the Malawi Developmental Assessment Tool (MDAT). A quantile g-computation model evaluated the linkage between multi-chemical exposures and developmental milestones. Of the 310 children in the follow-up, a majority had at least one form of developmental impairment (50.7 %; n=157) across four domains: gross motor (20.3%), fine motor (23.9%), language (28.3%), and social skill (16.2%). Increased Pb, Hg, Cd, and As exposure jointly reduced gross motor by 17.78% (aPR= 0.822; 95% CI: 0.6994, 0.966). Further, joint exposure to these heavy metals decreased language ability by 55.36% (aPR=0.446; 95% CI: 0.313, 0.636) and decreased general developmental milestones by 13.36% (aPR=0.866; 95% CI: 0.747,1.005). However, the combined effect on the fine motor (aPR=0.943; 95% CI: 0.754, 1.180) and social skills 6.71% (aPR=1.067; 95% CI: 0.694, 1.641) were not statistically significant. Exposure to heavy metals while in utero reduced children's neurodevelopmental milestones at 3-4 years of age. The cumulative impact of Pb, Hg, Cd, and As was significant for gross motor, language ability, and general impairment. The independent effects of Pb and Hg were amplified beyond what would be expected under the additive assumption with Cd and As, suggesting synergistic effects.
**Additional Information:**	
**Question**	**Response**
**Financial Disclosure**	The Department of Environmental and Occupational Health at the Catholic University of Health and Allied Sciences partially funded the fieldwork and laboratory work.

Powered by Editorial Manager® and ProduXion Manager® from Aries Systems Corporation

Enter a financial disclosure statement that describes the sources of funding for the work included in this submission and the role the funder(s) played. This includes grants and any commercial funding of the work or authors.<br><br>This statement will be typeset if the manuscript is accepted for publication.<br><br>*Please review the submission guidelines and the instructions link below for detailed requirements and guidance.*	
**Competing Interests**<br><br>On behalf of all authors, disclose any competing interests that could be perceived to bias this work.<br><br>This statement will be typeset if the manuscript is accepted for publication.<br><br>*Please review the instructions link below and PLOS Global Public Health's competing interests policy to determine what information must be disclosed at submission.*	There is no conflict of interest that may compromise the result of the result
**Data Availability**<br><br>Before publication, Authors are required to make fully available and without restriction all data underlying their findings. Please see our PLOS Data Policy page for detailed information on this policy.<br><br>A **Data Availability Statement**, detailing where the data can be accessed, is required at first submission. Insert your Data Availability Statement in the box below.	De-identified data and detailed information regarding the participants are available, See S1_DID

Powered by Editorial Manager® and ProduXion Manager® from Aries Systems Corporation

Please see the data reporting section of our submission guidelines for instructions on what you need to include in your Data Availability Statement.<br><br>This statement will be typeset if the manuscript is accepted for publication.<br><br>*PLOS allows rare exemptions to address legal and ethical concerns. If you have legal or ethical restrictions, please use the box below to detail these in full sentences for the Journal team to consider.*	

Powered by Editorial Manager® and ProduXion Manager® from Aries Systems Corporation

Manuscript
Click here to access/download;Manuscript;Manuscript_Chemical Mixture.docx

Title Page

Effects of Prenatal Lead, Mercury, Cadmium, and Arsenic Exposure on Children's Neurodevelopment in an Artisanal Small-scale Gold Mining Area in Northwestern Tanzania Using a Multi-chemical Exposure Model

Elias C. Nyanza[1], Raphael J. Mhana[2], Moses Asori[3], Deborah S.K. Thomas[3], and Agapiti P. Kisoka[4]

1 Department of Environmental, Occupational, and Research GIS, School of Public Health, Catholic University of Health and Allied Sciences, Mwanza, TANZANIA

2 Reproductive and Child Health Unit, Mwanza Regional Health Services, Mwanza, TANZANIA

3 Department of Geography and Earth Sciences, University of North Carolina at Charlotte, Charlotte, North Carolina 28223, USA

4 Social and Environmental Development, Kahama, TANZANIA

*****Correspondence: Elias C. Nyanza, elcnyanza@gmail.com

Effects of Prenatal Lead, Mercury, Cadmium, and Arsenic Exposure on Children's Neurodevelopment in an Artisanal Small-scale Gold Mining Area in Northwestern Tanzania Using a Multi-chemical Exposure Model

Abstract

An estimated 200 million children under five fail to reach their cognitive development potential in Africa. Growing evidence suggests reduced neurodevelopment for children from environmental exposures, yet research on this topic in Sub-Saharan Africa remains limited. This study examined the effects of multi-chemical prenatal exposure to heavy metals on developmental milestones for children aged 3-4 in artisanal and small gold mining areas in northwestern Tanzania. This longitudinal follow-up study of children whose mothers were enrolled in the *Tanzania Mining and Health Cohort* in Geita District in 2015 was assessed at 3-4 years of age for the current study between June 2019 – June 2020. Developmental outcomes (cognitive, social, motor, and language skills) were assessed using the Malawi Developmental Assessment Tool (MDAT). A quantile g-computation model evaluated the linkage between multi-chemical exposures and developmental milestones. Of the 310 children in the follow-up, a majority had at least one form of developmental impairment (50.7 %; n=157) across four domains: gross motor (20.3%), fine motor (23.9%), language (28.3%), and social skill (16.2%). Increased Pb, Hg, Cd, and As exposure jointly reduced gross motor by 17.78% (aPR= 0.822; 95% CI: 0.6994, 0.966). Further, joint exposure to these heavy metals decreased language ability by 55.36% (aPR=0.446; 95% CI: 0.313, 0.636) and decreased general developmental milestones by 13.36% (aPR=0.866; 95% CI: 0.747,1.005). However, the combined effect on the fine motor (aPR=0.943; 95% CI: 0.754, 1.180) and social skills 6.71% (aPR=1.067; 95% CI: 0.694, 1.641) were not statistically significant. Exposure to heavy metals while in utero reduced children's neurodevelopmental milestones at 3-4 years of age. The cumulative impact of Pb, Hg, Cd, and As was significant for gross motor, language ability, and general impairment. The independent effects of Pb and Hg were amplified beyond what would be expected under the additive assumption with Cd and As, suggesting synergistic effects.

Key Words: Neurodevelopmental; prenatal exposure; chemical mixture; gold mining; children

1. Introduction

Exposure to chemicals contributes to an estimated 4.9 million deaths and 86 million disabilities globally every year, representing approximately 25% of the disease burden worldwide (1). An estimated 200 million children under five in Sub-Saharan Africa do not reach full cognitive development potential (1). From a life course perspective, developmental impairment in early childhood diminishes future educational and occupational opportunities and negatively impacts social and economic mobility (2,3). By extension, cognitive decline across large swaths of the population restricts the potential for regional and national development (1,3,4).

Environmental exposure in utero and the early years of life increases the risk for physical- and mental health challenges later in adulthood (2,5). Recent evidence reveals that even the previous generation's exposure can have negative health consequences in later years (6,7). Exposure to chemicals in utero and early childhood has been associated with neurocognitive and psychomotor developmental impairments in earlier and later childhood (6–9); with an estimated 25% of early childhood developmental impairments attributed to environmental exposures (6). In particular, the adverse health effects of arsenic (As), cadmium (Cd), lead (Pb), and mercury (Hg) are well documented even at low doses in early fetal life (6,7,10–12), although the burden is not as well studied in Sub-Saharan Africa.

Exposure to non-essential metallic elements is shaped by human activities, including artisanal and small-scale gold mining (ASGM), which is relatively common in most African countries (13–15). ASGM activities are a primary source of environmental pollution/contamination, particularly Hg as a result of gold amalgamation, and As, Pb, and Cd as constituents of most gold ore (14,16,17). Studies conducted in ASGM areas in northwestern Tanzania revealed that soil, water sources, cassava leaves, and roots are contaminated with several chemical elements, including As, Hg, Pb, and Cd (14). Human exposure to these heavy metals occurs through inhalation of polluted air, drinking contaminated water, and consuming food/crops prepared/grown in contaminated soil or irrigated with contaminated water (14,18).

Exposure to toxic chemical elements is cumulative and compounding (Kasprzyk-Hordern et al., 2021; Pécheux et al., 2022; Willey et al., 2021). This may induce synergistic or intensifying health risks beyond an additive effect. For instance, even though Pb reduced the mental development index among children, the effect intensified with Hg and Cd (22). A similar synergistic effect of Pb in the presence of Hg and polychlorinated biphenyls is reported between

neurophysiological disorders and Pb exposure among children in Canada (23). While chemical-specific studies linking one chemical to a health outcome have provided helpful information to inform regulatory policies, such studies fail to fully reveal the nature of multi-chemical exposures when individuals are exposed to multiple chemicals simultaneously or sequentially (24). Improved models that more fully capture the simultaneity of heavy metals exposures can provide more robust evidence for policy.

Pregnant women in artisanal and small-scale gold mining (ASGM) areas are exposed to relatively high levels of chemicals (7, 25). Evidence indicates dose-related associations between exposure to toxic chemical elements and developmental delays in children (6, 7, 12, 26). However, there is some inconsistency in findings, with some studies reporting no association between prenatal exposure and early child developmental impairment (27,28), while others find significant associations (29,30). Some of the variability could be due to interaction and compounding effects.

The current study examined the effects of multi-chemical prenatal exposure on neurodevelopmental milestones for children aged 3-4 years born to mothers in the *Tanzania Mining and Health Cohort* in ASGM areas in northwestern Tanzania. The hypothesis is that Pb, Hg, As, and Cd will have synergistic or intensifying impacts on neurodevelopmental ability under joint exposure.

2. Materials and Methods

2.1. Study design, study setting, and study population

This is a longitudinal follow-up study as part of the *Tanzania Mining and Health Cohort* in Geita District in northwestern Tanzania established in 2015 in ASGM areas. The details of this cohort have been explained elsewhere (25,31). Briefly, the cohort included 883 pregnant women who were followed through to document the pregnancy outcomes. All children aged 3-4 years whose mother's chemical element levels were part of the *Tanzania Mining and Health Cohort* and still residing in the study area were recruited into the current study undertaken from 30th June 2019 to 25th May 2020. Mothers and their children in the cohort were excluded if they moved out of the ASGM area for over six months. A total of 352 mother-child pairs were recruited in this follow-up study, of which 310 were eligible, consented, and participated (Fig 1).

2.2. Ethical approval and consent to participate

The joint Catholic University of Health and Allied Science and the Bugando Medical Center Ethics and Research Review Committee reviewed and approved the study protocol (CREC/038/2014, CREC/379/2019, and CREC/699/2023). Permission to conduct the research was obtained from Geita Regional and District Council local authorities. Written informed consent was obtained from mothers who participated in the study. For children below 5 years participation, individual mothers signed a special assent form permitting the assessment of their children's neurodevelopmental status. Individual mothers had an opportunity to read and listen to the purpose, benefits, and risks of the research along with the rights of the participants before being asked for consent. Individual children who were sick during the assessment were directed to seek medication at a nearby health facility and hence were excluded from the study. Mothers had an opportunity to know the developmental state of their children and benefited from the comprehensive counseling on early stimulation as a way to mitigate the impact of developmental delays.

2.4. Data collection and statistical analysis

Parental data on socio-demographic and socio-economic characteristics of the caregivers (e.g., occupation, economic status, age, marital status, parity) and demographic characteristics of the child (e.g., sex, age, number of siblings in a family) were collected using a face-to-face structured interview questionnaire carried out at their homes. The economic status (SES) of the family was determined using a socioeconomic wealth quintile based on traditional indicators of wealth possession at the household level as detailed elsewhere (25,31), similar to those used in *Tanzanian Demographic and Health Surveys* (32). Households that scored ≥9 on the SES assessment were classified as high-SES families; those that scored 6-9 were considered moderate SES families, while those <6 were considered low-SES families (25). As co-variates in the regression analysis, children's characteristics, including age, sex, parental education, and family socio-economic status, were descriptively analyzed, and a univariate regression analysis was conducted to exclude all covariates with *p*-value >0.2 (two tails) using *Stata version 14.1* (*Stata Corp LP*®, College Station, Texas, USA).

Data on mothers' chemical element levels were utilized from the *Tanzania Mining and Health Cohort* (25, 31). Details are provided elsewhere (25, 31). Chemical levels were estimated using dried blood spots (DBS) for Hg, Cd, and Pb and urine samples for As (25, 31, 33). Spot

urine samples were collected in acid-washed polyethylene containers preserved with 1% hydrochloric acid and stored below -8°C before shipment to the laboratory. This was to prevent bacteria growth and absorption of the analytes. A drop of capillary blood was collected on filter paper following a simple finger prick for Cd, Pb, and Hg. Field and laboratory blanks (i.e., blank Whatman #903 filter papers) were included in sampling and analytical procedures to account for possible contamination and to avoid exposure misclassification. The DBS and urine samples underwent microwave-assisted closed vessel acid digestion to extract chemical element analytes from DBS and urine at 170°C for 30 minutes. These analytes were determined by inductively coupled plasma mass spectrometry (ICP-MS; PerkinElmer, Shelton, CT, USA) (μg/L) at an ISO 17025 accredited laboratory (ALS Scandinavia, Sweden). Apart from prenatal exposure levels, postnatal exposure among the children enrolled in this study was not considered.

Children's neurodevelopmental milestones were assessed using the *Malawi Developmental Assessment Tool* (MDAT), which has been developed and validated in low-income countries and is an appropriate tool for assessing children aged between 0-6 years (34). The MDAT has been translated into Swahili the common language in Tanzania, validated in our local settings – and used in the *Tanzania Mining and Health Cohort* (7). The MDAT contains 136 items, with 34 items across four domains (gross motor, fine motor, language, and social). The neurodevelopmental assessment was conducted by trained public health nurses blinded to the children's prenatal exposure concentrations and was determined based on the total score (ranging from 0 to 34 for each domain) (7). Children who performed ≥ 90th percentile on all of the items in that domain or < 90th percentile on one or two items in that domain were classified as usual (34). Those who performed < 90th percentile on more than two items in a domain were classified as impaired. In addition, individual children were classified in terms of global neurodevelopment status. In contrast, those who scored normal outcomes on all domains were classified as typically developing, and those impaired on at least one domain were classified as impaired for neurodevelopmental outcomes (7). In the present study, the median motor scores were 11 (IQR=9-13), of which the minimum was 4 and the maximum was 15, while the alpha coefficient for the items was 0.89. The median language scores were 7 (IQR=6-7), with a minimum score of 4 and a maximum of 20, while the alpha coefficient for the items was 0.91. The median social functioning score was 10 (IQR=8-11), of which the minimum was 5, and the maximum was 19,

with an alpha coefficient of 0.94. In all domains, the alpha coefficient suggests good internal consistency.

Assessing the joint effects of multiple toxic chemical exposures on children's developmental milestones is challenging with traditional regression models because: 1) they are unable to account for internal correlation/multicollinearity between chemical elements, and 2) they are unable to optimize each chemical’s weight or contribution on the overall joint effect. As a result, we used the quantile g-computation approach. Even though weighted quantile sum (WQS) regression has been used to make causal inferences for multi-chemical exposures on health outcomes, forcing chemical weights to have unidirectional effects (positive direction) in the WQS has been critiqued for introducing bias and inconsistency in model parameters. Therefore, proposed a more flexible and robust g-computation approach to address previously identified challenges in joint exposure modelling with the WQS model has been recommended (35). For the dose-response relationship of joint exposure using quantile g-computation, parameters are estimated as:

$$Y_i = \beta_0 + \psi \sum_{j=1}^{d} w_j X_{ji}^{q} + \varepsilon_i = \beta_0 + \psi \sum_{j=1}^{d} \beta_j X_{ji}^{q} + \varepsilon_i \quad (\textit{Equation ....1})$$

Where: X_{ji}^{q} is the quantized version of exposure X_j, ψ is the weight given as $\sum_{j=1}^{d} \beta_j$ (where β_j is the effect size of exposure j).

The sum of all weights $\psi = 1$; therefore, the individual weight for each heavy metal is given as:

$$w_k = \frac{\beta_k}{\sum_j^d \beta_j} \quad (\textit{Equation ....2})$$

Where: w_k is the weight for each chemical element, whereby weights can take on negative or positive directions based on the dose-response pattern between the health outcome and the exposure under consideration. Since our dependent variable was binary for each of the development components, we estimated the model $\Pr(Y = 1|X^d)$ with logistic regression and then converted to log (RR) (calculated automatically by the *g-comp* package in *RStudio*).

Three separate models were built: (1) the multi-chemical model, where all the chemicals were entered but unadjusted for confounding; (2) the multi-chemical model, where all the chemicals were entered and adjusted for confounding; and (3) a chemical-specific model; where one chemical was entered at a time and adjusted for confounding effect. An anti-logarithm of the log (RR) was used to convert it to a prevalence ratio (PR) for straightforward interpretation. We calculated 95% confidence intervals (CIs) around point estimates with 1000 bootstrap

resampling iterations with Monte-Carlo simulations to address uncertainties introduced from the sample size effect. Relationships were significant at a 5% level. All modelling exercises were conducted with the *qcomp* package in *RStudio version 4.3.2* (36).

3. Results

Table 1 summarizes the characteristics of parents and children. The median age of mothers was 25 (IQR=20-30), and 69.7% were between 20 and 34 years. Most mothers (80.3%; n=249) had at least a primary school education. Based on asset ownership assessment, 91% (n=282) had moderate and high SES. Most of the mothers (n=244; 78.7%) were either farming or keeping animals and only nine mothers (2.9%) were in mining. Among fathers, more than a quarter (n=144; 46.5%), were working in mining. The average age of children was 3.7 (SD=0.3) years and more than half, 225 (51.4%) were females.

Table 2 summarizes maternal prenatal chemical element levels from the initial study of the *Tanzania Mining and Health Cohort* in the current study. The total median urinary As level was 8.3 (IQR=4.5-14.9) µg/L; more than three-thirds (n= 234; 75.4%) of mothers had less than 15µg/L of urinary As (Table 3). The median total level of blood Hg was 1.2 µg/L (IQR=0.8-1.9) among mothers, and the majority (n= 237; 76.5%) had higher than ≥0.8 µg/L. The median prenatal Cd levels were 0.20 (IQR=0.2-0.3) µg/L, with the majority (n=261; 84.3%) of mothers having Cd levels <0.30 µg/L. Mothers' median prenatal blood Pb levels were 27.2 µg/L (IQR=17.2-42.5). Over one-third of children were prenatally exposed to Pb levels above 35 µg/L.

The prevalence of neurodevelopmental impairment among the study participants was above 15% across all domains. Specifically, 20.3%, 23.9%, 28.3, and 16.2% of the participating children were impaired in their neurodevelopmental status for gross motor, fine motor, language ability, and social interaction ability, respectively. Additionally, for global neurodevelopmental status, slightly above half (50.6%) of the participating children were impaired in at least one of the neurodevelopmental domains.

The association between multi-chemical exposures and the five domains of cognitive developmental outcomes (fine motor, gross motor, social functioning, language ability, and

general neurodevelopmental impairment) are reviewed in the following sections. Figures 2 and 3 below show the joint exposure effects for the five domains.

Fig 2 (Insert Fig 2 here)

Fig 3 (Insert Fig 3 here)

One quantile increase in the Pb, Hg, Cd, and As joint exposure reduced the global neurodevelopmental performance by 13.34% (aPR=0.866; 95% CI: 0.747,1.005; *p*=0.047) (Table 3). In a chemical-specific model, Pb reduced global neurodevelopmental performance by 10.5% (aPR= 0.895, 95% CI: 0.810- 0.989; *p*= 0.030). However, in a mixed-effect model, the Pb level significantly decreased global neurodevelopmental performance by 23.8% (aPR=0.762; *p*=0.02) (Table 4).

In the chemical-specific model, Hg reduced global neurodevelopmental performance by 7% (aPR=0.930, 95% CI: 0.853-1.014; *p*=0.10) (Table 5), whereas in a mixed-model, Hg (aPR=0.8446) decreased it by 15.6. Furthermore, compared to boys, girls had a 39.3% (aPR=0.607; *p*= 0.040) decline in global neurodevelopmental performance. Also, a unit change in age was statistically significantly associated with a 6.1% increase in global neurodevelopmental performance (aPR=1.061; *p*=0.015). The mother's level of education had no protective effect, even though, compared to those without formal education, those with ordinary education levels (aPR=0.308; *p*=0.017) had a nearly 71% decline in global neurodevelopmental performance.

Pb, Hg, Cd, and As jointly reduced gross motor ability by 17.8% (aPR= 0.822; 95% CI: 0.699, 0.966; p=0.041) (Table 3). In a chemical-specific model, Pb reduced gross motor ability by 11.5% (aPR=0.885, 95% CI: 0.793- 0.988; *p*= 0.030), while in a mixed model, Pb decreased gross motor by 22.5% (aPR=0.7752, *p*=0.028) (Table 4). Similarly, Hg reduced gross motor ability by 8.8% in a chemical-specific model (aPR=0.912, 95% CI: 0.824- 1.010; *p*= 0.078) (Table 5), and 16.8% (aPR=0.832; *p*=0.094) in the mixed model. As reduced gross motor ability by 4.8% (aPR=0.952, 95% CI: 0.858-1.056; *p*=0.354) in single-element exposure, while in the mixed model, it reduced gross motor ability by 7% (aPR=0.930; *p*=0.518). Additionally, children born to parents with ordinary education levels decreased their gross motor performance by 79.83% (aPR=0.201; *p*=0.002). Furthermore, age was statistically significantly related to gross motor ability (aPR =1.054; *p*= 0.035).

Although not statistically significant, Pb, Hg, and As levels jointly negatively affected fine motor performance (aPR=0.763), while Cd produced a positive effect (aPR=1.188) (Table 3). Even though no association was found, Pb still made the most significant contribution as a risk factor (45.9%), followed by Hg (42.3%) and As (11.8%). Children from low SES backgrounds had a 62.7% decrease in fine motor performance compared to higher SES (aPR=0.373; *p*=0.015) (Tables 4).

The combined effects of all the chemical elements decreased language ability by 55.4% (aPR=0.446; 95% CI: 0.313, 0.636; *p*<0.001) (Table 3). In a chemical-specific model, Pb reduced language ability by 43.9% (aPR=0.561, 95% CI: 0.434-0.726; *p*<0.001) while in a mixed model, it decreased language ability by 49.1% (aPR=0.509; *p*<0.0001) (Table 4). Furthermore, individually, Hg reduced language ability by 22.8% (aPR=0.772, 95% CI: 0.621-0.959; *p*=0.019) (Table 5), while in the mixed model, Hg decreased language ability by 21.9% (aPR=0.781; *p*=0.084). Cd (aPR= 0.777, 95% CI: 0.620- 0.973; *p*= 0.028) significantly reduced language ability by 22.3%, but the association was insignificant in a mixed model (Cd: aPR=0.865; *p*=0.344). Additionally, sex (aPR=0.926; *p*=0.810), mother's primary school education level (aPR=0.837; *p*=0.645), the child's age (aPR=1.0519; *p*=0.185), moderate SES (aPR=1.711; *p*=0.141), and low SES (aPR=1.345; *p*=0.520) were not statistically significantly related to language ability.

Although not statistically significant, the joint effect of all the chemical elements was positively associated with social functioning at 6.7% (aPR=1.067; 95% CI: 0.694, 1.641; p>0.10) (Table 3). While the combination of Pb and Cd had a positive joint impact (aPR=1.585), Hg and As provided a joint antagonistic effect (aPR=0.682), thus nullifying the overall combined effect. However, in the negative direction, prenatal exposure to Hg had the highest weight (99.78%), followed by As (0.225%), while in the positive direction, Cd had the most significant weight (60.5%), followed by Pb (39.5%). Even though no association was observed for either the chemical-specific or mixed model, Hg had a potentiation effect, while other elements remained relatively unchanged.

4. Discussion

Fifty percent of the children in this current study had neurodevelopmental impairment in one or more domains, which is higher than when these children were assessed (31%) in infancy (7). Children developed additional impairments at three to four years, providing evidence that

neurodevelopmental deficits from prenatal exposures continue later in life (30). The increasing severity and prevalence of neurodevelopmental impairment among children as they age could occur for a variety of reasons. Firstly, brain development begins earlier in embryonic life and continues well beyond childhood into adulthood (37,38). During these early stages, the brain cells undergo mitosis and migrate, differentiate, develop synaptic connections, and undergo programmed apoptosis (38). Therefore, any interference from toxic exposures may alter subsequent developmental milestones (37,39). Even a slight disruption from Pb and Hg at a fetal or infancy stage may continue to have long-term cascade effects later in life, including learning deficits (40). Secondly, prenatally exposed children may continue to be exposed to even higher environmental pollutants after birth. Exposure at the community level also occurs from home, play, and travel, including to-from and at school (41–43). Drinking contaminated water or eating soil from the ground may add to the existing health burden carried by the prenatal exposure stages (43).

Generally, Pb and Hg appeared to induce a toxic effect on neurodevelopmental abilities among children. Significantly, when interacting with other chemical elements, their individual marginal effects are amplified beyond what would be expected under the additive assumption. Given the magnitude of neurodevelopmental impairment may increase as the child grows and even worsens when the child is multi-chemically exposed, stringent environmental control measures that limit toxic exposures are needed. Further, setting new safety reference levels that integrate multi-chemical exposure scenarios is necessary.

Joint exposure to Pb, As, Hg, and Cd increased the risk of neurodevelopmental impairment in one or more domains (the global neurodevelopmental impairment). While blood Pb and Hg were statistically significantly related to global neurodevelopmental impairment, Pb carried the most significant weight in the mixed model (contributing to about 59.3% of the total weight), suggesting its dominant role in inducing neurodevelopmental impairment. Compared to those with no formal education, children whose mothers had ordinary-level education had about a 70% reduced global neurodevelopmental ability. This trend suggests the need to consider how sociodemographic disparities and gender differences interact with multi-chemical exposures to elevate neurodevelopmental impairment among children.

Combined Pb, As, Cd, and Hg exposure was linked to decreased gross motor ability. Accounting for confounding effects, Pb independently reduced gross motor. In the presence of

other chemicals, the negative impact of Pb on gross motor ability was higher than expected under the additive assumption. Our findings corroborate other international studies. For instance, prenatal exposure to Pb in the third trimester significantly decreased the development index of infants at 6 months of age (41). In a Tanzanian study, Pb and As independently did not induce neurodevelopmental impairment among infants. However, when they acted in the presence of Hg, there was a potentiated outcome (7). This may be explained by the neurotoxic effect of Pb on nerve cells (44). Cell damage in the hippocampal and volt sections of the brain due to Pb exposure may affect execution function, including a decrease in gross motor function. Further, since Hg, Cd, and As may have similar biokinetic pathways for inducing toxicity, the marginal effect of Pb may be amplified when exposed jointly with other heavy metals. Clinical evidence suggests that the combined impact of Pb, Cd, and As can elevate neural oxidative stress by increasing the levels of Ca^{2+} (45). They can also jointly stimulate extracellular signal-regulated kinase (ERK), c-Jun N-terminal kinases (JNK), and mitogen-activated protein kinase3 (MAPK3) pathways, which may cumulatively affect various neurological and psychomotor functions in children (45). Even though Pb independently reduced the mental development index as reported among South Korean children, the effect amplified when it interacted with Hg and Cd (22). A similar synergistic effect of Pb in the presence of Hg and polychlorinated biphenyls has been found between neurophysiological disorders and Pb exposure among children in Montreal, Canada (23).

Elevated Hg levels were linked to decreased gross motor function, accounting for approximately 36% of the total risk weight. Even though Hg independently reduced gross motor, the impact was higher in the presence of other metallic elements, suggesting a synergistic effect. For example, a quartile increase in Hg was linked to an 8.78% decrease in gross motor ability and about 16.8% when Hg interacted with Pb, Cd, and As. In a previous study from the US, poor psychomotor development among children at 6 months was associated with elevated prenatal Hg exposure (46). The synergistic effect of Hg in the presence of Pb and Cd is equally reported in a study investigating cognitive development and multi-chemical exposures in South Korea (22). Methylmercury, Pb, and As have similar neurological pathways, including disrupting the expression of N-methyl-D-aspartate (NMDA) receptors (47). Therefore, their individual effects may be more than additive when exposed in combination. We did not, however, find any association between Cd and As and gross motor ability. This may be explained by the restrictive

passage of Cd through the placenta from the protein metallothionein (48,49). Nevertheless, we found that their impact was elevated in the presence of Hg and Pb, suggesting they may also have potentiating effects. More studies are therefore needed to provide concrete evidence better.

Although the association was not statistically significant, Pb, Hg, As, and Cd reduced fine motor ability in multi-chemical and single-element models. Similarly, no association was found between heavy metals exposure and social interaction in single and multiple-chemical models. In the social interaction model, besides Hg, which was inversely linked, we observed a positive association with other heavy metals. This effect direction contradicts other studies' reported effect direction (45,50,51), although it is possible the association in the current study was due to random chance.

The combined effect of Pb, As, Hg, and Cd was statistically significantly associated with decreased language ability, with Pb playing a significant role in the joint effect. Independently, the impact of Pb was synergistic, as the risk of language ability decline increased from 43% in single-element exposure to 49% in multi-chemical exposure. In addition to the reductive effect of Pb exposure on IQ scores, memory, social interaction, and deficits in language ability are observed among children exposed to Pb contamination (45). Exposure to Pb is highly damaging to the central nervous system (CNS) (46). Pb exposure affects the CNS's dopaminergic, cholinergic, and glutamatergic systems. For instance, an abnormal release of dopamine transporters is linked to Pb exposure, which can increase language and communication difficulties (45,52). Additionally, the cholinergic system known to regulate energy metabolism can be affected by Pb exposure (45,53).

In a recent study, children who resided in Pb-contaminated neighborhoods had smaller volumes of the mid-anterior and mid-posterior corpus callosum (54). The decline in these systems affected processing and language speed (54). In a large *National Health and Nutrition Examination Survey (NHANES)* study, about 1,025,695 people in the U.S. who were born between 1989 and 1998 and had difficulties in listening, reading, reasoning, or speaking were exposed to elevated Pb levels (55). Similarly, exposure to >22.2ppm of Pb was linked to an 8.6-point decrease in word identification test compared to those exposed to 5.99ppm (45). As the primary target may involve the CNS, the health consequences may be long-term extending from infancy to adulthood. This may explain why about 50% of children in our current study had neurodevelopmental impairment in at least one of the four domains, compared to 31% during

infancy. The effects may worsen as the child grows and gets exposed to multiple pollutants from diverse sources.

Hg exposure significantly reduced language ability in single-element and multi-chemical exposure models. Children with an increased level of Hg in their hair, fingernails, and toenails are more likely to score lower in the Bayley-III scale score of expressive language (56). Other studies support this evidence (57–59). Our results also support a study in Spain where prenatal Hg exposure has been associated with poor performance in verbal function at 4 to 5 years of age (58). However, in the reactive presence of other heavy metals, a decreased risk of Hg exposure, from 54% risk to 22% was discovered. This may be due to different elements' antagonistic effects in the joint model. Even though a quartile increases in Cd and As were associated with a 4% and 14% decrease in language ability, respectively, they were not statistically significant. This is supported by the finding in the Korean population study among children, where early prenatal Cd exposure had no significant association with cognitive and psychomotor developmental impairment, even with increased Pb levels at 6 months of age (60). Similar findings have been reported in the US *National Health and Nutrition Examination Survey* (NHANES) (61). Since this study considered a marginal population-level effect, we recommend that future studies focus on sub-group studies to better examine the complexities of these linkages. Our findings suggest that existing safety thresholds, predominantly based on single-element studies, would need to be modified to account for the joint exposure effect from heavy metals and other environmental pollutants. Secondly, considering that the health effects may worsen as the child grows due to the cumulative impact of pre-and postnatal exposures, there is likely a need to establish community–based early childhood development interventions in areas with ASGM activities. The need to provide young children with cognitively stimulating activities and expose them to learning opportunities and better activity spaces is crucial. The current study provides evidence that supports a call for action to reduce environmental pollution associated with ASGM activities in affected areas. There is a need to promote the implementation of early childhood stimulation programs to minimize the impact of developmental impairments among children residing in ASGM areas.

The findings that the individual marginal effects of Pb and Hg on neurodevelopmental abilities are amplified beyond what would be expected under the additive assumption when reacted with other chemical elements calls for strengthening the growing demand to modify

existing health risk reduction strategies, which are predominantly based on single-element assessment. Adapting existing safety thresholds in the context of multi-chemical exposures, which is a reality for women and children in gold mining areas, may be timely. While the combined effects of these heavy metals may lead to synergistic impacts, socioeconomic disparities, and gender likely play an interactive role. This underscores the need to incorporate subgroup modeling into the multi-chemical exposure assessment framework.

This study is limited to prenatal exposure among children born to mothers in ASGM communities. This follow-up nested study does not consider exposures to chemical elements post-delivery. The actual amount of the toxic chemical elements transferred to the fetus may vary from one woman to another depending on maternal health status (62). Future bio-monitoring studies should consider maternal health status and individual children's exposure status. Recruitment was limited for consistency to mother-child pairs who stayed in the ASGM area to follow up on neurodevelopment. Children were assessed at a time when they were not sick or in need of any medical assistance. The individual children's percentile scores in each domain during the assessment, apart from the passing or failing of a child at the 90% percentile level for their age, might have reduced the precision of the results. However, using a validated African-developed tool (i.e., the MDAT) provides robustness to the findings and accounts for the local settings. The adequate sample size and sampling of appropriate bio-indicators (i.e., maternal prenatal blood for Hg, Pb, and Cd; and urine for As) (62) provide value to the integrity of the findings.

5. Conclusion

Children born to women in areas with ASGM activities who are prenatally exposed to Pb, Hg, and As are at increased risk for neurodevelopmental impairment. The cumulative impact of all chemicals was significant for gross motor, language ability, and general impairment. The magnitude of neurodevelopmental impairments increases as children grow. The independent effects of Pb and Hg were amplified beyond what would be expected under the additive assumption with Cd and As, suggesting synergistic or potentiating effects. Neurodevelopmental health risks among children varied according to gender, educational, and socioeconomic status (SES), drawing attention to the need for studies focused on environmental health inequities in the African context.

Declarations

Availability of data and materials: De-identified data and detailed information regarding the participants are available as supporting information S1.

Conflicts of Interest: The authors declared that there are no direct or indirect competing interests that exist.

Funding: Partial funding for the fieldwork and laboratory work was received from the Department of Environmental and Occupational Health at the Catholic University of Health and Allied Sciences.

Author Contributions: ECN contributed to the study conceptualization and design, supervised the data collection, contributed to the statistical analysis, interpreted the results, and assisted in writing the manuscript. RJM contributed to the study conceptualization and design, supervised the data collection, and interpreted the results. MA contributed to the statistical analysis, interpretation of the findings, and writing of the manuscript. DSKT contributed to the study conceptualization, statistical analysis review, interpretation of the findings, and drafting and writing the manuscript. AP contributed to the study conceptualization and design and supervised the data collection. All authors read and approved the final draft of the manuscript

Acknowledgments: The authors acknowledge support and assistance from the Department of Environmental and Occupational Health at the Catholic University of Health and Allied Sciences and the Department of Geography and Earth Sciences at the University of North Carolina at Charlotte. The authors would like to thank all the healthcare workers, women, and children participating in the *Tanzania Mining and Health Cohort* for participating in this study.

References

1. Grantham-Mcgregor S, Cheung YB, Cueto S, Glewwe P, Richter L, Strupp B. Child development in developing countries 1 Developmental potential in the fi rst 5 years for children in developing countries [Internet]. Vol. 369, Lancet. 2007. p. 60–70. Available from: www.thelancet.com
2. Black MM, Walker SP, Fernald LCH, Andersen CT, DiGirolamo AM, Lu C, et al. Early childhood development coming of age: science through the life course. The Lancet. 2017 Jan 7;389(10064):77–90.
3. Jackson MI. A Life Course Perspective on Child Health, Academic Experiences and Occupational Skill Qualifications in Adulthood: Evidence from a British Cohort. Soc Forces Sci Medium Soc Study Interpret. 2010;89(1):89–116.
4. Bauer JA, Devick KL, Bobb JF, Coull BA, Bellinger D, Benedetti C, et al. Associations of a metal mixture measured in multiple biomarkers with IQ: Evidence from italian adolescents living near ferroalloy industry. Environ Health Perspect. 2020 Sep;128(9):097002-1-097002–12.
5. Scattolin MA de A, Resegue RM, Rosário MC do. The impact of the environment on neurodevelopmental disorders in early childhood. J Pediatr (Rio J). 2022 Apr 20;98:66–72.
6. Golding J, Gregory S, Iles-Caven Y, Hibbeln J, Emond A, Taylor CM. Associations between prenatal mercury exposure and early child development in the ALSPAC study. NeuroToxicology. 2016 Mar;53:215–22.
7. Nyanza EC, Bernier FP, Martin JW, Manyama M, Hatfield J, Dewey D. Effects of prenatal exposure and co-exposure to metallic or metalloid elements on early infant neurodevelopmental outcomes in areas with small-scale gold mining activities in Northern Tanzania. Environ Int. 2021 Apr;149.
8. Grandjean P, Landrigan PJ. Neurobehavioural effects of developmental toxicity. Vol. 13, The Lancet Neurology. 2014. p. 330–8.
9. Klus JK, Thurston SW, Myers GJ, Watson GE, Rand MD, Love TM, et al. Postnatal methylmercury exposure and neurodevelopmental outcomes at 7 years of age in the Seychelles Child Development Study Nutrition Cohort 2. NeuroToxicology. 2023 Dec;99:115–9.
10. Bellinger DC, Matthews-Bellinger JA, Kordas K. A developmental perspective on early-life exposure to neurotoxicants. Vol. 94, Environment International. Elsevier Ltd; 2016. p. 103–12.
11. Debes F, Budtz-Jørgensen E, Weihe P, White RF, Grandjean P. Impact of prenatal methylmercury exposure on neurobehavioral function at age 14 years. Neurotoxicol Teratol. 2006;28(3):363–75.
12. Gao Y, Yan CH, Tian Y, Wang Y, Xie HF, Zhou X, et al. Prenatal exposure to mercury and neurobehavioral development of neonates in Zhoushan City, China. Environ Res. 2007 Nov;105(3):390–9.
13. Msoffe CU, Nyanza EC, Thomas DSK, Jahanpour O, Dewey D. The sources and chemical content of edible soil sticks sold in markets in Tanzania: a cross-sectional analytical study. Environ Geochem Health. 2019;41(2):893–906.
14. Nyanza EC, Joseph M, Premji SS, Thomas DSK, Mannion C. Geophagy practices and the content of chemical elements in the soil eaten by pregnant women in artisanal and small scale gold mining communities in Tanzania. BMC Pregnancy Childbirth. 2014 Apr;14(1).
15. Taylor BA, Casas-Ganem J, Vaccaro AR, Hilibrand AS, Hanscom BS, Albert TJ. Differences in the work-up and treatment of conditions associated with low back pain by patient gender and ethnic background. SPINE. 2005;30(3):359–64.
16. Steckling N, Tobollik M, Plass D, Hornberg C, Ericson B, Fuller R, et al. Global Burden of Disease of Mercury Used in Artisanal Small-Scale Gold Mining. 2016.
17. Taylor H, Appleton JD, Lister R, Smith B, Chitamweba D, Mkumbo O, et al. Environmental assessment of mercury contamination from the Rwamagasa artisanal gold mining centre, Geita District, Tanzania. Sci Total Environ. 2005;343(1–3):111–33.
18. Taylor H, Appleton JD, Lister R, Smith B, Chitamweba D, Mkumbo O, et al. Environmental assessment of mercury contamination from the Rwamagasa artisanal gold mining centre, Geita District, Tanzania. Sci Total Environ. 2005 May;343(1–3):111–33.
19. Kasprzyk-Hordern B, Proctor K, Jagadeesan K, Lopardo L, O'Daly KJ, Standerwick R, et al. Estimation of community-wide multi-chemical exposure via water-based chemical mining: Key research gaps drawn from a comprehensive multi-biomarker multi-city dataset. Environ Int. 2021 Feb;147.

20. Pécheux M, Saoudi A, Zeghnoun A, Oleko A, Fillol C. Assessment of multi-chemical exposure using human biomonitoring data from the French Esteban study using exposure load method. Vol. 246, International Journal of Hygiene and Environmental Health. Elsevier GmbH; 2022.
21. Willey JB, Pollock T, Thomson EM, Liang CL, Maquiling A, Walker M, et al. Exposure Load: Using biomonitoring data to quantify multi-chemical exposure burden in a population. Int J Hyg Environ Health. 2021 May;234.
22. Shah-Kulkarni S, Lee S, Jeong KS, Hong YC, Park H, Ha M, et al. Prenatal exposure to mixtures of heavy metals and neurodevelopment in infants at 6 months. Environ Res. 2020 Mar;182.
23. Boucher O, Burden MJ, Muckle G, Saint-Amour D, Ayotte P, Dewailly É, et al. Response inhibition and error monitoring during a visual Go/No-Go task in Inuit children exposed to lead, polychlorinated biphenyls, and methylmercury. Environ Health Perspect. 2012 Apr;120(4):608–15.
24. Hernández AF, Tsatsakis AM. Human exposure to chemical mixtures: Challenges for the integration of toxicology with epidemiology data in risk assessment. Vol. 103, Food and Chemical Toxicology. Elsevier Ltd; 2017. p. 188–93.
25. Nyanza EC, Bernier FP, Manyama M, Hatfield J, Martin JW, Dewey D. Maternal exposure to arsenic and mercury in small-scale gold mining areas of Northern Tanzania. Environ Res. 2019 Jun;173:432–42.
26. Choi AL, Grandjean P. Methylmercury exposure and health effects in humans. Environ Chem. 2008;5(2):112–20.
27. ChingChun LCL, YuChuan CYC, FengChiao SFS, ChienMu LCL, HuaFang LHL, YawHuei HYH, et al. In utero exposure to environmental lead and manganese and neurodevelopment at 2 years of age. 2013;
28. Myers GJ, Davidson PW, Cox C, Shamlaye CF, Palumbo D, Cernichiari E, et al. Prenatal methylmercury exposure from ocean fish consumption in the Seychelles child development study. The lancet. 2003;361(9370):1686–92.
29. Grump KS, Kjellström T, Shipp AM, Silvers A, Stewart A. Influence of prenatal mercury exposure upon scholastic and psychological test performance: Benchmark analysis of a New Zealand cohort. Risk Anal. 1998;18(6):701–13.
30. Kippler M, Tofail F, Hamadani JD, Gardner RM, Grantham-McGregor SM, Bottai M, et al. Early-life cadmium exposure and child development in 5-year-old girls and boys: A cohort study in rural Bangladesh. Environ Health Perspect. 2012 Oct;120(10):1462–8.
31. Nyanza EC, Dewey D, Manyama M, Martin JW, Hatfield J, Bernier FP. Maternal exposure to arsenic and mercury and associated risk of adverse birth outcomes in small-scale gold mining communities in Northern Tanzania. Environ Int. 2020 Apr;137.
32. TNBS-MIS. TNBS-MIS (Tanzania National Bureau of Statistics- Malaria Indicator Survey). Tanzania Demographic and Health Survey and Malaria Indicator Survey (TDHS-MIS) - Final Report 2015-16. 2017.
33. Nyanza EC, Dewey D, Bernier F, Manyama M, Hatfield J, Martin JW. Validation of dried blood spots for maternal biomonitoring of nonessential elements in an artisanal and small-scale gold mining area of Tanzania. Environ Toxicol Chem. 2019;38(6):1285–93.
34. Gladstone M, Lancaster GA, Umar E, Nyirenda M, Kayira E, Broek NR van den, et al. The Malawi Developmental Assessment Tool (MDAT): The creation, validation, and reliability of a tool to assess child development in rural African settings. PLoS Med. 2010 May;7(5).
35. Keil AP, Buckley JP, O'Brien KM, Ferguson KK, Zhao S, White AJ. A quantile-based g-computation approach to addressing the effects of exposure mixtures. Environ Health Perspect. 2020;128(4).
36. Keil A. The qgcomp package: g-computation on exposure quantiles. The qgcomp package: g-computation on exposure quantiles. 2023.
37. Bellinger DC. Prenatal Exposures to Environmental Chemicals and Children's Neurodevelopment: An Update. Saf Health Work. 2013 Mar 1;4(1):1–11.
38. Schettler T. Toxic threats to neurologic development of children. Environ Health Perspect. 2001 Dec;109(suppl 6):813–6.
39. Makri A, Goveia M, Balbus J, Parkin R. CHILDREN's SUSCEPTIBILITY TO CHEMICALS: A REVIEW BY DEVELOPMENTAL STAGE. J Toxicol Environ Health Part B [Internet]. 2004 Nov 1 [cited 2024 Jul 19]; Available from: https://www.tandfonline.com/doi/full/10.1080/10937400490512465
40. Harari R, Julvez J, Murata K, Barr D, Bellinger DC, Debes F, et al. Neurobehavioral Deficits and Increased Blood Pressure in School-Age Children Prenatally Exposed to Pesticides. Environ Health Perspect. 2010 Jun;118(6):890–6.

41. Landrigan PJ, Baker EL. Exposure of children to heavy metals from smelters: Epidemiology and toxic consequences. Environ Res. 1981 Jun 1;25(1):204–24.
42. Ngo HTT, Watchalayann P, Nguyen DB, Doan HN, Liang L. Environmental health risk assessment of heavy metal exposure among children living in an informal e-waste processing village in Viet Nam. Sci Total Environ. 2021 Apr;763.
43. Parlak M, Tunçay T, Botsou F. Heavy Metals in Soil and Sand from Playgrounds of Çanakkale City (Turkey), and Related Health Risks for Children. Sustainability. 2022 Jan;14(3):1145.
44. Dribben WH, Creeley CE, Farber N. Low-level lead exposure triggers neuronal apoptosis in the developing mouse brain. Neurotoxicol Teratol. 2011 Jul;33(4):473–80.
45. Goel A, Aschner M. The effect of lead exposure on autism development. Vol. 22, International Journal of Molecular Sciences. MDPI AG; 2021. p. 1–12.
46. Marques RC, Dórea JG, Bastos WR, Rebelo M de F, Fonseca M de F, Malm O. Maternal mercury exposure and neuro-motor development in breastfed infants from Porto Velho (Amazon), Brazil. Int J Hyg Environ Health. 2007 Feb;210(1):51–60.
47. Vuong AM, Yolton K, Braun JM, Lanphear BP, Chen A. Chemical mixtures and neurobehavior: A review of epidemiologic findings and future directions. Vol. 35, Reviews on Environmental Health. De Gruyter Open Ltd; 2020. p. 245–56.
48. Caserta D, Graziano A, Monte GL, Bordi G, Moscarini M, Caserta D. Heavy metals and placental fetal-maternal barrier: a mini-review on the major concerns. Eur Rev Med Pharmacol Sci. 2013;17(16).
49. Kippler M, Hoque AMW, Raqib R, Öhrvik H, Ekström EC, Vahter M. Accumulation of cadmium in human placenta interacts with the transport of micronutrients to the fetus. Toxicol Lett. 2010 Feb;192(2):162–8.
50. Mendelsohn AL, Benard ;, Dreyer P, Fierman AH, Rosen CM, Legano LA, et al. Low-Level Lead Exposure and Behavior in Early Childhood [Internet]. Vol. 101. 1998. Available from: http://www.pediatrics.org/cgi/content/full/101/3/
51. Roy A, Bellinger D, Hu H, Schwartz J, Ettinger AS, Wright RO, et al. Lead exposure and behavior among young children in Chennai, India. Environ Health Perspect. 2009 Oct;117(10):1607–11.
52. Chao OY, Pathak SS, Zhang H, Dunaway N, Li JS, Mattern C, et al. Altered dopaminergic pathways and therapeutic effects of intranasal dopamine in two distinct mouse models of autism. Mol Brain. 2020 Aug;13(1).
53. Balfour JL, Kaplan GA. Neighborhood Environment and Loss of Physical Function in Older Adults : Evidence from the Alameda County Study. 2002;155(6):507–15.
54. Marshall AT, McConnell R, Lanphear BP, Thompson WK, Herting MM, Sowell ER. Risk of lead exposure, subcortical brain structure, and cognition in a large cohort of 9-to 10-year-old children. PLoS ONE. 2021 Oct;16(10 October).
55. Geier DA, Kern JK, Geier MR. Blood lead levels and learning disabilities: A cross-sectional study of the 2003-2004 national health and nutrition examination survey (NHANES). Int J Environ Res Public Health. 2017 Oct;14(10).
56. Hsi HC, Jiang CB, Yang TH, Chien LC. The neurological effects of prenatal and postnatal mercury/methylmercury exposure on three-year-old children in taiwan. Chemosphere. 2014;100:71–6.
57. Freire C, Ramos R, Lopez-Espinosa MJ, Díez S, Vioque J, Ballester F, et al. Hair mercury levels, fish consumption, and cognitive development in preschool children from Granada, Spain. Environ Res. 2010 Jan;110(1):96–104.
58. Freire C, Amaya E, Gil F, Fernández MF, Murcia M, Llop S, et al. Prenatal co-exposure to neurotoxic metals and neurodevelopment in preschool children: The Environment and Childhood (INMA) Project. Sci Total Environ. 2018 Apr;621:340–51.
59. Surkan PJ, Wypij D, Trachtenberg F, Daniel DB, Barregard L, McKinlay S, et al. Neuropsychological function in school-age children with low mercury exposures. Environ Res. 2009 Aug;109(6):728–33.
60. Kim JH, Jeong KS, Ha EH, Park H, Ha M, Hong YC, et al. Association between prenatal exposure to cadmium and atopic dermatitis in infancy. J Korean Med Sci. 2013;28(4):516–21.
61. Ciesielski T, Weuve J, Bellinger DC, Schwartz J, Lanphear B, Wright RO. Cadmium exposure and neurodevelopmental outcomes in U.S. children. Environ Health Perspect. 2012;120(5):758–63.
62. Tratnik JS, Falnoga I, Trdin A, Mazej D, Fajon V, Miklavčič A, et al. Prenatal mercury exposure, neurodevelopment and apolipoprotein E genetic polymorphism. Environ Res. 2017 Jan;152:375–85.

Table 1: Parental Socio-demographic and socio-economic characteristics

Parental Baseline characteristics	N	(%)
Maternal median age years (25, IQR: 20 -30)		
Maternal age group*		
14-24	107	(34.5)
25-34	148	(47.7)
35+	55	(17.7)
Mother education		
No formal	61	(19.7)
At least primary school	249	(80.3)
Social Economic Wealth Quintile (SEWQ)		
Good or High (>9)	211	(68.1)
Moderate (6-9)	64	(20.6)
Low (<6)	35	(11.3)
Mother's occupation		
Farm/Animal keeping	244	(78.7)
Mining	9	(2.9)
Business and public servant	57	(18.4)
Father occupation		
Farm/Animal keeping	98	(31.6)
Mining	144	(46.5)
Business & Public Servant	68	(21.9)
Marital Status		
Married	285	(91.9)
Single	5	(1.6)
Separated	19	(6.1)
Widow	1	(0.3)
Relationship with partner		
Good and supportive	284	(91.6)
Poor (single-parent care)	23	(7.4)
Separated (child with grandparent)	3	(1)
Individual Children Characteristic		
Mean age of children (43.9±3.4) months		
Sex of child		
Male	163	(52.7)
Female	147	(47.3)
Presence of under-fives at home		
Yes	346	(78.8)
No	93	(21.2)

Table 2: Maternal prenatal blood total mercury (Hg), blood total Cadmium (Cd), blood total lead (Pb), and total urinary arsenic (As) (N=310)

Prenatal Exposure Levels	N	(%)	P10	P25	Median (95%CI)	P75	P95	Max
Total As (µg/L)			2.8	4.5	8.3(7.4-9.3)	14.9	38.2	213
≥ 15	76	(24.6)			-			
<15	234	(75.4)			-			
Total Mercury (µg/L)			0.6	0.8	1.2 (1.1-1.3)	1.7	3.8	72
≥0.8	237	(76.5)			-			
<0.8	73	(23.4)			-			
Total Cadmium (µg/L)			0.14	0.21	0.2(0.2-0.21)	0.3	0.5	1.5
≥0.3	49	(15.7)						
<0.3	261	(84.3)						
Total Lead (µg/L)			11.6	17.2	27.2(25-30.3)	42.5	85.8	145.1
<35	194	(62.6)						
≥35	116	(37.4)						

Note: *We dichotomized Pb, Hg, Cd, and As based on human biomonitoring reference values established by the GerESIV of 35.0 µg/L, 0.80 µg/L, 0.30 µg/L for blood lead, mercury, and cadmium, and 15 µg/L for urinary arsenic (Schulz et al. 2009). CI, confidence interval; ug/l, P, percentile; ug/l, microgram/liter.*

Table 3: Unadjusted and covariate-adjusted model on chemical elements mixture effects on neurodevelopmental abilities

**Unadjusted Model**					
	General Impairment cPR (95% Lower CI, Upper CI)	*Social Behavior* cPR (95% Lower CI, Upper CI)	*Language Ability* cPR (95% Lower CI, Upper CI)	*Gross Motor* cPR (95% Lower CI, Upper CI)	*Fine Motor* cPR (95% Lower CI, Upper CI)
Intercept	0.646(0.522, 0.800)	0.118(0.0542, 0.2571)	0.504(0.352, -0.324)	0.644(0.519, 0.799)	0.4454(0.3137, 0.6325)
psi1	0.8811(0.759, 1.023) †	1.149(0.728, 1.815)	0.418(0.302, 0.579) ***	0.811(0.6843, 0.961)*	0.943 (0.7540, 1.1801)
Cd+As	1.172	1.642 (Cd+Pb+As)	0.307 (Pb+Hg+Cd)	0.612 (Pb+Hg+As)	0.763 (Pb+Hg+As)
Pb + Hg	0.645	0.717(Mercury)	1.009(Arsenic)	1.074(Cd)	1.188(Cd)
Pb weight	0.686(-)	0.350(+)	0.632(-)	0.658(-)	0.509(-)
As weight	0.275 (-)	0.027(+)	1(+)	0.330(-)	0.0321(-)
Cd weight	0.725 (+)	0.623 (+)	0.143(-)	0.012(-)	1(+)
Hg weight	0.314(-)	1(-)	0.225(-)	1(+)	0.459(-)
**Covariate Adjusted Model**					
	General Impairment aPR (95% Lower CI, Upper CI)	*Social Behavior* aPR (95% Lower CI, Upper CI)	*Language Ability* aPR (95% Lower CI, Upper CI)	*Gross Motor* aPR (95% Lower CI, Upper CI)	*Fine Motor* aPR (95% Lower CI, Upper CI)
(Intercept)	0.659(0.545, -0.225)	0.132(0.0635,0.2752)	0.463(0.313, 0.684)	0.633(0.512, 0.784)	0.459(0.329, 0.640)
psi1	0.866(0.747,1.01) *	1.067(0.694, 1.641)	0.446(0.313, 0.636) ***	0.822(0.699, 0.966) *	0.923(0.739, 1.153)
Pb+As+Hg	0.632	1.586 (Pb+Cd)	0.3296(all)	0.600	0.735
Cd	1.125	0.682 (Hg+As)	---------	1.095	1.182
Pb weight	0.593(-)	0.395(+)	0.610(-)	0.499(-)	0.459(-)
As weight	0.036(-)	0.002(-)	0.036(-)	0.142(-)	0.118(-)
Cd weight	1(+)	0.605(+)	0.131(-)	1(+)	1(+)
Hg weights	0.371(-)	0.998(-)	0.223(-)	0.359(-)	0.423(-)

Note: *† = significant at 0.1; *=significant at 0.05; **=significant at 0.001; ***= significant at 0.0001; psi1=combined effects of all chemical mixers; neg=negative weight; pos=positive weight; CI =confidence interval; aPR=adjusted prevalence ratio; cPR=crude Prevalence ratio.*

Table 4: Marginal effects of individual chemicals and associated covariates on neurodevelopmental abilities

**Prenatal Exposure Characteristics**	**General Impairment** aPR	**Social Interactions** aPR	**Language Ability** aPR	**Gross Motor** aPR	**Fine Motor** aPR
(Intercept)	*0.584*	*0.008*	*0.145*	*1.584*	*0.507*
Pb	0.762*	1.1998	0.509***	0.775*	0.868
As	0.984	0.999	0.961	0.930	0.964
Cd	1.125	1.322	0.866	1.095	1.182
Hg	0.844	0.683	0.781†	0.833†	0.878
Sex					
Boys	ref				
Girls	0.607*	0.716	0.927	0.737†	0.868
Birth Weight	*0.819*	*1.122*	*0.903*	*0.627*	*0.993*
Maternal Education					
No education	ref				
Primary school	0.807	1.196	0.837	0.730	0.605
Ordinary level	0.308**	0.426	0.158†	0.202**	0.461
Tertiary	0.736	4.438E-07	1.68E-06	0.863	0.829
Child age	*1.061***	*1.067*	*1.052*	*1.054**	*1.030*
SES status					
High	ref				
Moderate	0.799	0.985	1.711	0.878	0.735
Low	0.495†	0.418	1.345	0.565	0.373*

Note: *†=significant at 0.10; *= significant at 0.05; **= significant at 0.001; ***= significant at 0.0001*

Table 5: Individual effects from the covariate-adjusted univariate model on neurodevelopmental abilities

	**General Impairment**	**Social**	**Language**	**Gross Motor**	**Fine Motor**
	aPR	aPR	aPR	aPR	aPR
Pb	0.895**	1.183	0.561***	0.885*	0.973
As	0.981*	1.0096	0.916	0.952	0.964
Cd	1.014	1.219	0.777*	1.000	1.066
Hg	0.930 †	0.784	0.459†	0.912†	0.935

Note: *†=significant at 0.10; *= significant at 0.05; **= significant at 0.001; ***= significant at 0.0001*

Figure
Click here to access/download;Figure;Figures.docx

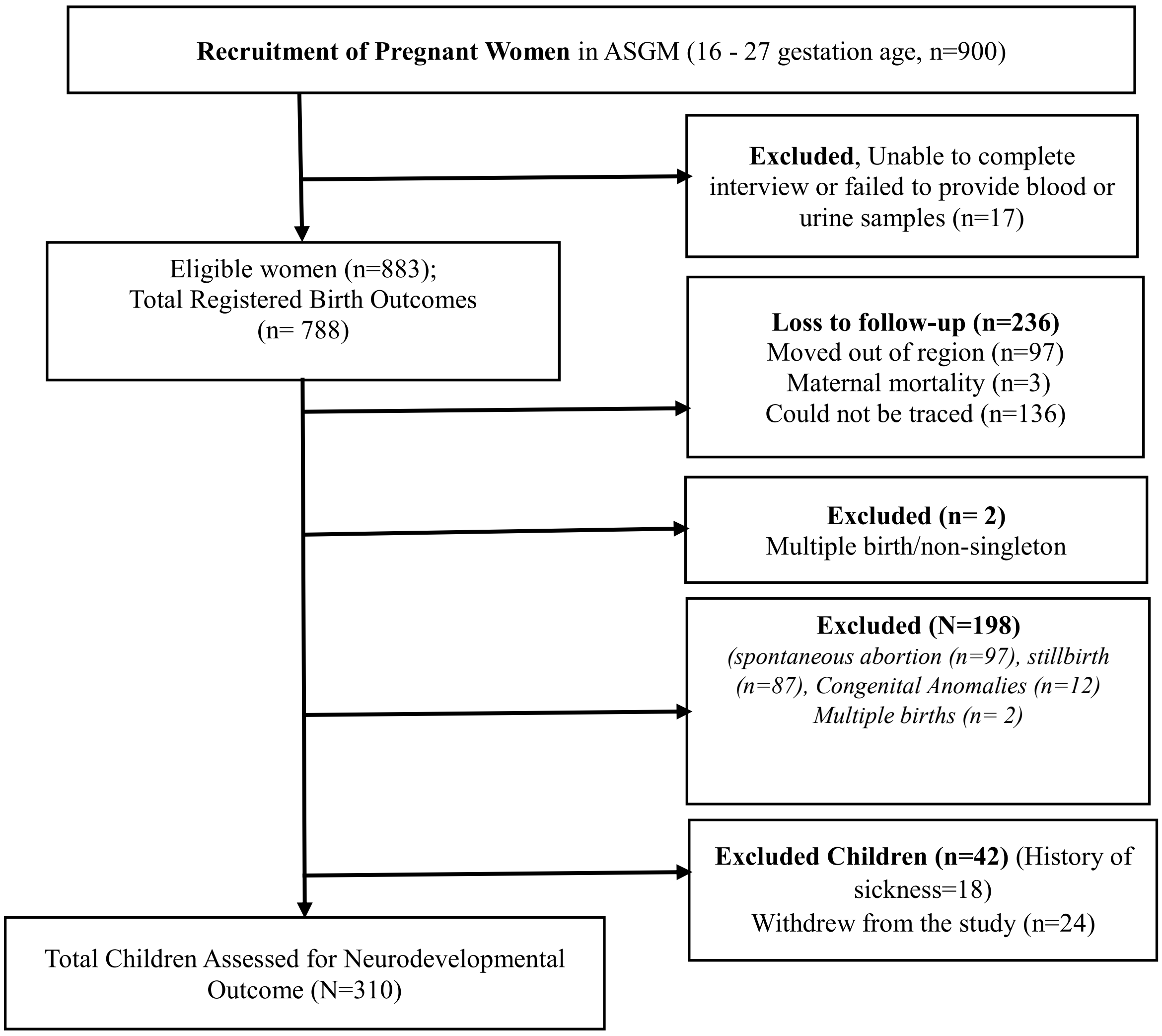

Fig 1. Recruitment, exclusion and eligibility, and loss to follow-up of mothers and children in ASGM areas.

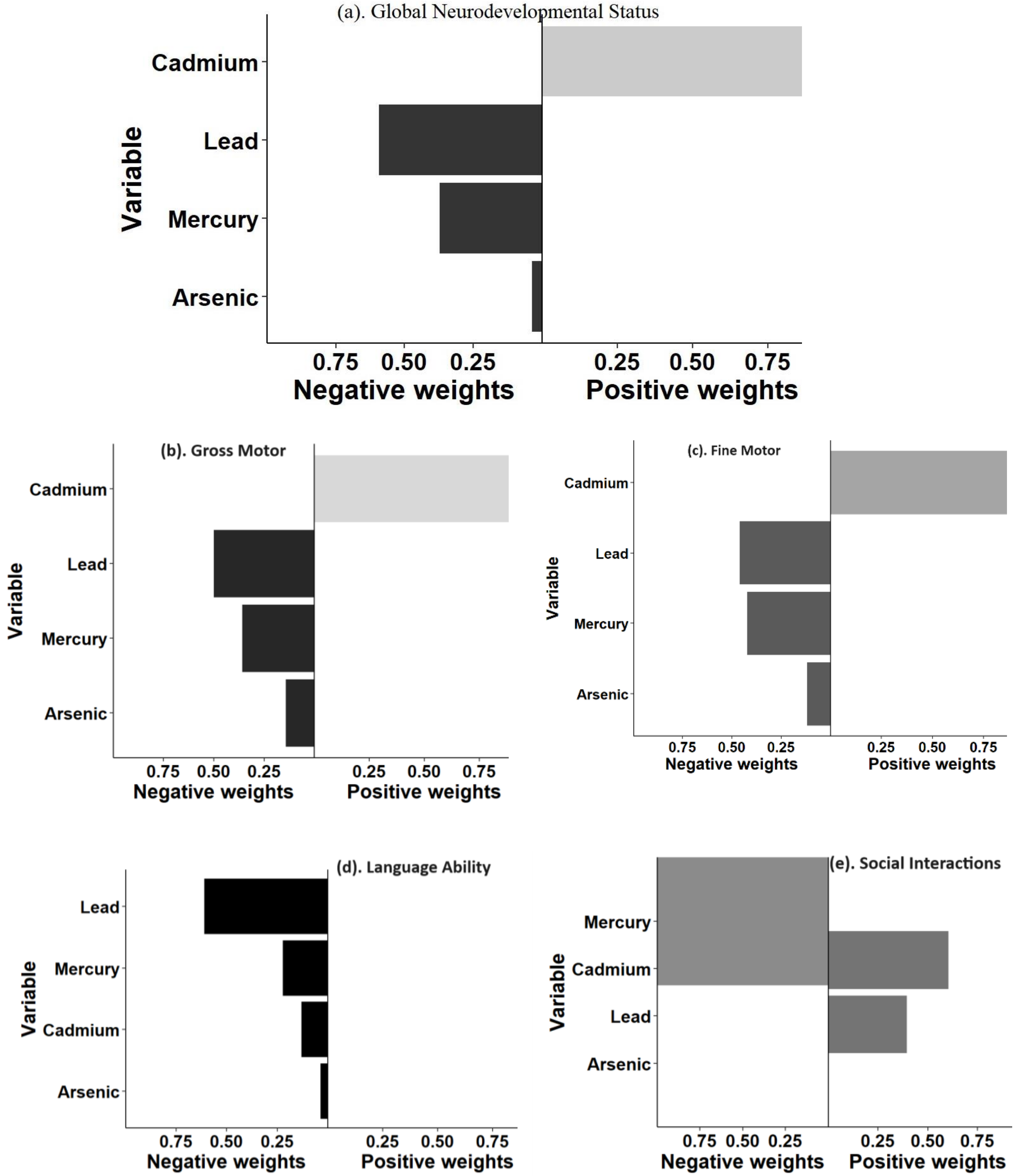

Figure 2: Effects of individual chemical elements and their weights on neurodevelopmental impairments in mixed interaction models. The bar plots pointing to the right represent chemicals that contributed positively (increased risk) to the joint exposure risk. In contrast, those pointing to the left indicate chemicals inversely contributing to the joint exposure risk.

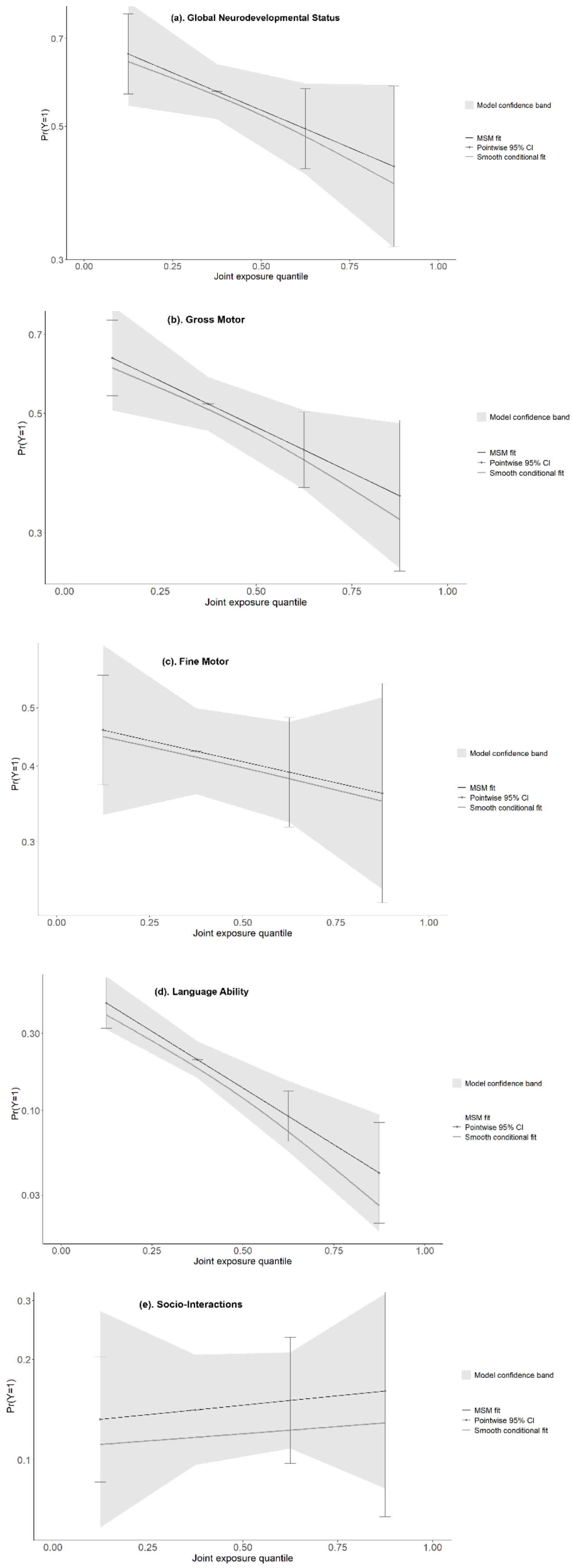

Fig 3: A pointwise 95% confidence interval of the model uncertainty band for (a) Global Neurodevelopmental status, (b). Gross Motor, (c). Fine Motor, (d). Language Ability and (e). Socio-interactions. The y-axis indicates the probability of not being cognitively impaired, while the x-axis shows the quantile distribution of the joint exposures. The MSM fit is an estimate from the marginal structural model.

Click here to access/download
Supporting Information
Inclusivity-in-global.docx

Click here to access/download
Supporting Information - Compressed/ZIP File Archive
STROBE-checklist-v4-combined-PlosMedicine_Chemical Mixture.docx

Click here to access/download
Supporting Information - Compressed/ZIP File Archive
S1_DID Data.sav
