# Supplementary material for: Effects of prenatal lead, mercury, cadmium, and arsenic exposure on children’s neurodevelopment in an artisanal small-scale gold mining area in Northwestern Tanzania using a multi-chemical exposure model
Source: PLOS Glob Public Health. 2025 Apr 30;5(4):e0004577. doi: 10.1371/journal.pgph.0004577 (PMC12043129; doi:10.1371/journal.pgph.0004577)
Supplement: Attachment — Submitted filename: Response to the Editor.docx [file pgph.0004577.s006.docx]

23.11.2024

Dear Editor,

**PLOS Global Public Health**

**Submission of a manuscript for Consideration in Publication in PLOS Global Public Health**

Please find attached the revised manuscript entitled “***Effects of Prenatal Lead, Mercury, Cadmium, and Arsenic Exposure on Children’s Neurodevelopment in an Artisanal Small-scale Gold Mining Area in Northwestern Tanzania using a Multi-chemical Exposure Model*** ” for Publication consideration in *PLOS Global Public Health*.

The current study examined the effects of multi-chemical prenatal exposure on neurodevelopmental outcomes for children aged 3-4 years born to mothers in the *Tanzania Mining and Health Cohort* in gold mining activities in northwestern Tanzania. **See Page 5, line 3 - 14.**

We have revised and indicated the ethics approval and written consent for the participating mothers and a special assent that was signed by the mothers for the participating children below 5 years.

Please address all correspondence about this manuscript to Dr. Elias C. Nyanza, [atelcnyanza@gmail.com](mailto:atelcnyanza@gmail.com)

I appreciate your handling of this paper.

Yours sincerely,

Dr. Elias C. Nyanza
