# Supplementary material for: Effects of prenatal lead, mercury, cadmium, and arsenic exposure on children’s neurodevelopment in an artisanal small-scale gold mining area in Northwestern Tanzania using a multi-chemical exposure model
Source: PLOS Glob Public Health. 2025 Apr 30;5(4):e0004577. doi: 10.1371/journal.pgph.0004577 (PMC12043129; doi:10.1371/journal.pgph.0004577)
Supplement: Attachment — Submitted filename: Response to the Editor_22.03.2025.docx [file pgph.0004577.s008.docx]

22.03.2025

Dear Editor,

**PLOS Global Public Health**

**Submission of a manuscript for Consideration in Publication in PLOS Global Public Health**

I am sharing with you the revised manuscript entitled “***Effects of Prenatal Lead, Mercury, Cadmium, and Arsenic Exposure on Children’s Neurodevelopment in an Artisanal Small-scale Gold Mining Area in Northwestern Tanzania using a Multi-chemical Exposure Model*** ” for Publication consideration in *PLOS Global Public Health*.

Responses to the editors’ queries;

1. Thank you for uploading your study's underlying data set, S1_DID. We noticed that this file may contain potentially identifying participant information in Column 1 titled ID and also the specific ages in the Age column. Please clarify whether the data in these fields are anonymized or need to be anonymized? If they do not need anonymization, please describe how they do not contain identifying information. If they do need further anonymization, then please remove or anonymize these columns from the file and re-upload your dataset in the interest of participant privacy. We will also remove the original file from our system.

Thank you for raising the concern and taking the initiative to protect participants. We have omitted the columns regarding the Identification number/code and the specific age for mothers and their participating children. The data in all fields are now anonymized.

2. Please amend your online detailed Financial Disclosure statement. This is published with the article. It must therefore be completed in full sentences and contain the exact wording you wish to be published.

The online Financial Disclosure Statement has been updated as directed.

a) Please clarify all sources of financial support for your study. List the grants, grant numbers, and organizations that funded your study, including funding received from your institution. Please note that suppliers of material support, including research materials, should be recognized in the Acknowledgements section rather than in the Financial Disclosure.
Financial support including grants have been described as directed.

b) State the initials, alongside each funding source, of each author to receive each grant. For example: ""This work was supported by the National Institutes of Health (####### to AM; ###### to CJ) and the National Science Foundation (###### to AM).""

This information has been added as directed.

c) State what role the funders took in the study. If the funders had no role in your study, please state: “The funders had no role in study design, data collection and analysis, decision to publish, or preparation of the manuscript.”
The funders had no role in the study design, data collection and analysis, decision to publish, or preparation of the manuscript.

If you did not receive any funding for this study, please simply state: “The authors received no specific funding for this work.””

3. Please ensure that the funders and grant numbers match in the Financial Disclosure field and the Funding Information tab in your submission form. The funders must also be provided in the same order in both places.

Where applicable, the funders and grant numbers have been matched between the Financial Disclosure field and the Funding Information tab in the same order.

4. Please update your online Competing Interests statement. If you have no competing interests to declare, please state: “The authors have declared that no competing interests exist.”

This information has been included as directed.

5. Please provide separate main figure files in .tif or .eps format only and ensure that all files are under our size limit of 10MB.

Figures have been saved in .tif within the size limit of 10MB.

6. Figures 2 and 3 are currently divided into multiple panels in separate files. Please include all panels for each figure within a single file, with each panel in the image labelled alphabetically (A, B, C). References to panels within figures should include the figure number and panel letter: e.g., Fig 1A, Fig 1B, Fig 1C. Alternatively, you may wish to separate panels into individual figures, renaming them accordingly (Fig 1A as Fig 1, Fig 1B as Fig 2, etc.).

Panels have been separated into individual figures as suggested.

7. Please add a full list of legends for all your Supporting Information files after the References list.

All supporting information included in this manuscript has been listed in the legend list after the references as directed.

I appreciate your handling of this paper.

Yours sincerely,

Dr. Elias C. Nyanza
